# Variable Neighborhood Search for Multi-Cycle Medical Waste Recycling Vehicle Routing Problem with Time Windows

**DOI:** 10.3390/ijerph191912887

**Published:** 2022-10-08

**Authors:** Wanting Zhang, Ming Zeng, Peng Guo, Kun Wen

**Affiliations:** 1 College of Management Science, Chengdu University of Technology, Chengdu 610059, China; 2 School of Mechanical Engineering, Southwest Jiaotong University, Chengdu 610031, China

**Keywords:** variable neighborhood search, medical waste recycling, vehicle routing problem, multi-cycle, time windows

## Abstract

Background: Improper disposal of urban medical waste is likely to cause a series of neglective impacts. Therefore, we have to consider how to improve the efficiency of urban medical waste recycling and lowering carbon emissions when facing disposal. Methods: This paper considers the multi-cycle medical waste recycling vehicle routing problem with time windows for preventing and reducing the risk of medical waste transportation. First, a mixed-integer linear programming model is formulated to minimize the total cost consisting of the vehicle dispatch cost and the transportation costs. In addition, an improved neighborhood search algorithm is designed for handling large-sized problems. In the algorithm, the initial solution is constructed using the Clarke–Wright algorithm in the first stage, and the variable neighborhood search algorithm with a simulated annealing strategy is introduced for exploring a better solution in the second stage. Results: The computational results demonstrate the performance of the suggested algorithm. In addition, the total cost of recycling in the periodic strategy is lower than with the single-cycle strategy. Conclusions: The proposed model and algorithm have the management improvement value of the studied medical waste recycling vehicle routing problem.

## 1. Introduction

With the rapid growth of the population and the rapid development of medical technology, the volume and composition of medical waste have grown rapidly over the past 30 years [1]. Especially since the outbreak of COVID-19 in early 2020, the amount of medical waste generated has increased dramatically [2]. For example, the amount of medical waste generated in Wuhan, China has increased from 40 tons/day before the epidemic to 110–150 tons/day [3], and the peak value was as high as 240 tons/day [4]. However, the gap between the production and disposal of medical waste is widening. Hence, the urgency of developing urban medical waste recycling is heightened [5]. Effective recycling of medical waste can prevent the spread of disease, reduce environmental pollution, and promote resource recycling [6]. Therefore, how to effectively recycle medical waste has become a hot issue for the people as well as the government [7]. The majority of medical waste is collected and disposed of centrally, and the transportation procedure is subject to stringent rules, including restrictions on the vehicle’s weight, the operating window, and other factors [8]. In addition, how to develop an environmentally conscious transportation network and reduce the carbon tax generated during the transportation of goods has also become one of the key issues [9]. Therefore, it is necessary for medical waste recycling to set up a rational and scientific path for recycling given these constraints.

The medical waste recycling problem can be regarded as a general situation of the vehicle routing problem with time windows (VRPTW), which is proved to be NP-hard [10]. Therefore, most of the current literature explores some efficient heuristic algorithms for the solution of VRPTW. Aylin et al. [11] investigated the medical waste recycling vehicle routing problem of Istanbul and comprehensively considered the factors of the recycling points, treatment points, transport vehicles, etc., so as to improve and optimize the recycling route of the medical waste and eventually determine the most feasible routes from the perspective of efficiency and economy. RFID technology was used by Nolz et al. [12] to optimize the planning process of predefined time horizons. Meanwhile, for the purpose of handling the randomness of the problem, adaptive large neighborhood search-based algorithms were also used to obtain the final vehicle route. Hachicha et al. [13] investigated the transport problem of medical waste from 12 hospitals in Tunisia. They considered it a route optimization problem and integrated the contagiousness of medical waste into load constraints. The real cases were then solved by interactive optimizer tool CPLEX. Mete et al. [14] proposed a geographic information system (GIS) solution approach to determine the optimal location of disposal centers, and the proposed approach was applied to minimize the total distance as well as the total risk during the transport of medical waste between 167 health facilities (collection centers) and five scheduled disposal centers through the TRB1 region of Turkey. Nikzamir et al. [15] proposed a bi-objective mixed-integer mathematical model for the transport of infectious and non-infectious medical waste problems by considering the factor of stochastic contamination emissions during the transport of infectious waste. Accordingly, a multi-objective class water flow algorithm with novel operators was developed to solve the problem, and the algorithm was thoroughly compared with the other algorithms. For the household expired medicine recycling vehicle routing problem, a traditional genetic algorithm was used by Wang [16] to improve the recycling rate. Vijayakumar et al. [17] focused on the bio-medical waste collection problem in Coimbatore, and the particle swarm optimization algorithm (PSO) was used to solve the mathematical model to minimize the total collection time. The safety score was applied by Eren et al. [4] to establish a linear programming model with two objective functions based on the safety score and the total transport distance. The model was then solved, and a mediation solution was obtained to determine the safest and shortest transport route for medical waste vehicles. Amirhossein et al. [18] proposed a novel waste management approach in real time by utilizing modern traceability devices of the Internet of Things (IoT), designed a two-stage system efficiently, and innovated the collection route in order to maximize the recycling value and achieve sustainable development. Due to home healthcare services and their associated demand rates increasing dramatically, Amin et al. [19] designed a home health care system based on the Internet of Things (IoT) and developed a two-step modeling approach to select a number of employed vehicles. Moreover, the green split pick-up vehicle routing problem (GSPVRP) and IoT concept are employed to address the patients in both sub-models. For the sake of solving the optimization problem of urban waste collection and transportation in China, Wu et al. [20] considered a green vehicle routing problem (PCGVRP) model in a waste management system, and the optimal solution is obtained by a local search hybrid algorithm (LSHA), and several instances are selected from the capacitated vehicle routing problem (CVRP) database so as to test and verify the effectiveness of the proposed LSHA algorithm. The literature survey has shown that a limited number of studies use vehicle routing models for solving the medical waste recycling problem, and there is also a lack of policy consideration regarding that medical waste must be fully recycled within 48 h [21]. There is no difference between the model of the existing research and the general network optimization model, so it is far away from the realistic background of medical waste recycling and does not reflect the characteristics of the medical waste recycling problem. How to serve specific medical institutions during specific time periods is the first question that needs to be addressed [22].

The periodic vehicle routing problem (PVRP) is a temporal extension of the VRP, first proposed by Beltrami and Bodin [23]. In PVRP, the optimal route is composed of different lines in multiple cycles, and customers need to visit multiple times during the cycle or have different requirements for the frequency of return visits [24], such as supermarket delivery, elevator maintenance, garbage collection, and letter delivery services [25]. Scholars have been looking for efficient methods to find the optimal solution or approximate optimal solution in the cycle, mainly including exact algorithm [26,27], heuristic algorithm [28,29], meta-heuristic algorithm, machine learning algorithm [30,31,32], and some more mature commercial software [33]. Compared with other algorithms, the meta-heuristic algorithm has a more comprehensive and thorough search process, which greatly improves the goodness of the solution and approximately solves a wide range of hard optimization problems without having to deeply adapt to each problem [34,35]. With these advantages, metaheuristic algorithms can be used to solve nonlinear, large-scale combinatorial optimization problems, covering engineering fields such as computers, automation, electronics, and intelligent robotics [36]. Therefore, scholars have generated rich research results in this neighborhood. For example, Hemmelmayr et al. [37] studied the problem of waste collection by considering the periodic vehicle routing problem with intermediate facilities (PVRP-IF). An exact dynamic programming formulation and an efficient hybrid solution based on varied neighborhoods were proposed. A set of benchmark examples were developed and searched. Cantu-Funes et al. [38] considered the periodic vehicle routing problem with additional factors, such as multiple models, multiple stations, and lead times. A stochastic adaptive greedy algorithm was then proposed for solving this problem. Alinaghian et al. [39] presented a new variant of the periodic vehicle routing problem in which reaching the customers affects the market share and where the objective function is to minimize the total transit time and maximize the market share. In order to solve this model, multi-objective particle swarm (MOPSO) and local MOPSO algorithms are applied, and the results of the algorithms are compared based on some comparison metrics. Racha et al. [40] proposed a meta-heuristic based on the particle swarm optimization (PSO) algorithm for the multi-period vehicle routing problem with profit (mVRPP) to maximize the total profit collected, where the planning horizon of each vehicle was divided into several cycles. Dong et al. [41] introduced a new model of PVRP, the periodic vehicle routing problem with flexible delivery dates, which covers a wider range of applications and is able to answer most diverse customer requirements. Additionally, they proposed an algorithm based on iterated local search (ILS). This metaheuristic approach involves an iterative application of a local search algorithm and the use of perturbation as a diversification mechanism. Chen et al. [42] studied a real-life container transportation problem with a wide planning horizon divided into multiple shifts; a variable neighborhood search algorithm with reinforcement learning (VNS-RLS) was thus developed. The study shows that algorithms can greatly reduce the rate of infeasible solutions explored during the search. Despite the achievements of meta-heuristics to obtain optimal solutions even for large-scale problems in a short period of time, novel and attractive improvements are emerging, improving the adaptability to the solution and stagnation prevention techniques [43]. The traditional algorithms, such as genetic algorithm, particle swarm optimization algorithm, variable neighborhood search algorithm, etc., have problems of slow solution speed and falling into a local optimum easily. Thus, this is another starting point of this research.

To answer these research questions, this study first explains the approaches to model and solve the problem we are concerned with. A mixed-integer linear programming model is designed for the multi-cycle medical waste recycling vehicle routing problem with time windows to reduce the number of vehicles dispatched and the transportation cost. Next, we propose an improved variable neighborhood search (IVNS) algorithm, which addresses the drawbacks of the conventional variable neighborhood search algorithm. The case study section presents the detailed results of the proposed decision optimization framework. We then examine the validity and superiority of the proposed metaheuristic-based approach for the multi-cycle medical waste recycling vehicle routing problem with time windows. The last section contains the conclusions and comments on limitations. To sum up, this study develops a decision-making framework to economically and efficiently determine the optimal routing of the medical waste recycling vehicle.

The contributions of this study are as follows:

(1) This is a pioneering study to establish a multi-cycle medical waste recycling vehicle routing decision-making framework with time windows.
(2) The decision-making framework developed takes into account the periodic strategy that can meet the demands for multi-frequency recycling services from medical institutions and improve the recycling efficiency of medical waste.
(3) An effective metaheuristic-based IVNS algorithm is proposed to solve the multi-cycle medical waste recycling vehicle routing optimization problem with time windows. A variety of retention strategies and improved perturbation mechanisms are devised to conquer the convergence rate and premature problem.

## 2. Problem Description and Formulation

**Figure 1:**
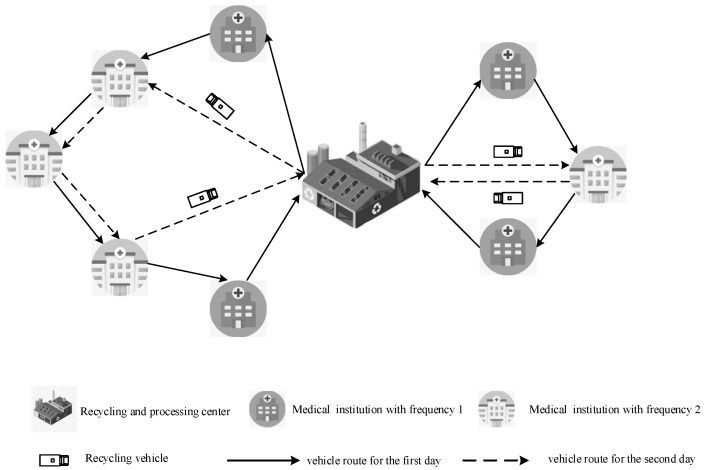
Example of a medical waste recycling problem.

The problem addressed in this paper can be summed up as follows: as depicted in Figure 1, the problem is that the medical waste recycling vehicles are dispatched from the recycling and processing center to the medical institutions on time to provide services for one-by-one recycling in accordance with the daily waste volume generated by the medical institutions and then return to the recycling and processing center to form a closed-loop process in the condition of the recycling and processing center. Two different types of decisions must be made in order to solve the medical waste recycling routing optimization problem using the cyclical strategy: (1) determining the service times for medical facilities based on the total number of services provided and the combination of available time windows; and (2) assigning vehicles and optimizing recycling routing for medical facilities within a given service time cycle. Medical facilities with a frequency of 2 are larger or produce more trash each day and must recycle every day of the cycle; facilities with a frequency of 1 can recycle any day of the cycle. There is typically only one medical waste recycling and processing facility in a city since medical waste is dangerous. The amount of medical waste generated each day fluctuates due to the different sizes and levels of medical facilities and the sporadic nature of the patients admitted each day.

With each medical waste recycling vehicle leaving the medical waste recycling and processing center (depot) via each recycling point, completing its recycling task for each customer point, and then returning to the recycling and processing center, it is assumed that the municipal medical waste recycling network consists of a medical waste recycling and processing center as well as several medical waste generation points (medical institutions). In addition, the subsequent assumptions are made:

(1) Medical facilities, recycling facilities, and processing facilities are located and counted.
(2) How much waste is recycled at each institution is known.
(3) The separation between nodes is the shortest distance traveling directly between two places.
(4) Only one truck per day is allowed to collect and transport the medical waste the healthcare facilities produce back to the facility for processing and recycling.
(5) The recycling vehicles for medical waste are of the same model and have a defined maximum vehicle capacity.
(6) For medical facilities, the recycling cycle lasts two days.
(7) Every vehicle and facility complies with the unique guidelines for moving and getting rid of medical waste.

Table 1 summarizes the notation for the mathematical model depicted in Figure 1.

According to the assumptions, a mixed-integer linear programming model is established as follows. The optimization objective is to minimize the total costs under the periodic recycling strategy, which includes the vehicles’ dispatch cost and transportation costs.

(1) $$\mathrm{Objective}\;\mathrm{function}:\;\min Z=\omega_{1}\times \sum_{t\in T}^{}\sum_{j\in N}y_{0,j}^{t}+\omega_{2}\times \sum_{t\in T}^{}\sum_{i\in N}^{}\sum_{j\in N}^{}\sum_{v\in V}^{}c_{ij}x_{ijv}^{t}$$

subject to:

(2) $$\sum_{i\in N_{0}}y_{iv}^{t}q_{i}/f_{i}\leq Q,\forall v\in V$$

(3) $$\sum_{t\in T}\sum_{v\in V}y_{iv}^{t}=\{\begin{array}{c} 2\sum_{v\in V}y_{iv}^{t},i=0,n+1\\ f_{i},i\in N_{} \end{array}$$

(4) $$\sum_{v\in V}y_{iv}^{t}\leq 1,\forall i\in N_{0}$$

(5) $$\sum_{i\in N}x_{ijv}^{t}=y_{iv}^{t},\forall j\in N_{0},t\in T,v\in V$$

(6) $$\sum_{j\in N}x_{ijv}^{t}=y_{iv}^{t},\forall i\in N_{0},t\in T,v\in V$$

(7) $$e_{i}\leq s_{i}^{v}\leq l_{i},\forall i\in N_{0},v\in V$$

(8) $$s_{i}^{v}+t_{ij}+se_{i}-s_{j}^{v}\leq (1-x_{ijv}^{t})•\psi ,\forall i,j\in N_{0},v\in V$$

(9) $$\sum_{j\in N}y_{0,j}^{t}\leq 1$$

(10) $$\sum_{i\in N}y_{i,n+1}^{t}\leq 1$$

(11) $$\sum_{i\in N}x_{ijv}^{t}-\sum_{j\in N}x_{ijv}^{t}=0,\forall p\in N,v\in V$$

(12) $$x_{ijv}^{t}\in \{0,1\},\forall i,j\in N_{0},v\in V,t\in t$$

(13) $$y_{\mathrm{iv}}^{t}\in \{0,1\},\forall i\in N_{0},v\in V,t\in t$$

The objective function (1) minimizes the total medical waste recycling vehicle dispatching cost and the transportation cost. Constraint (2) ensures the actual load of the medical waste recycling vehicle cannot exceed the maximum load. Constraint (3) expresses the number of times a medical institution is visited in a cycle equal to its required recycling frequency. Constraint (4) guarantees the medical institution *i* can be visited at most once a day. Constraints (5) and (6) require that there is at most one and only one medical waste recycling vehicle that carries out the recycling at each point on day *t*. Constraint (7) guarantees the medical waste recycling vehicle must visit the medical institution within the specified time window. The vehicle must not arrive at the medical institution later than the latest arrival time and must wait if it arrives earlier than the minimum value of the time window. Constraint (8) calculates the sum of the vehicle arrival time at medical institution *i*, the service time at medical institution *i*, and the traveling time from medical institution *i* to medical institution *j* of the vehicle cannot be later than the maximum value of the time window of medical institution *j*. Constraint (9) ensures the medical waste recycling vehicle must depart from the vehicle parking point 0 at the recycling and processing center on day *t*. Constraint (10) expresses the medical waste recycling vehicle must return to the vehicle parking point *n* + 1 at the recycling and processing center on day *t*. Constraint (11) is a traffic flow conservation constraint that ensures the vehicle *v* must leave the medical institution *i* after visiting. Constraints (12) and (13) define the domains of the decision variables.

## 3. Improved Variable Neighborhood Search Algorithm (IVNS)

A two-stage approach is designed to solve the multi-cycle medical waste recycling vehicle routing problem with time windows in this section. In the first stage, the initial solution is constructed by using the Clarke–Wright algorithm (C–W), while, in the second stage, the problem is solved by an improved variable neighborhood search algorithm combined with the idea of a simulated annealing algorithm. First proposed by Clarke and Wright [44] in 1964, the C–W algorithm is a greedy algorithm, the basic idea of which is to merge two loops of the transportation problem into one loop in turn so as to maximize the reduction in the total transportation distance at each time until the load limit of one vehicle is reached and then optimize the next vehicle. The variable neighborhood search algorithm is an improved local search algorithm that Mladenović first put forward in 1997 [45]. This algorithm can perform an alternative search through the neighborhood structures composed of different operations and achieve a good balance between concentration and sparsity. Meanwhile, the simulated annealing algorithm was proposed by N. Metropolis et al. [46] in 1953, and then S. Kirkpatrick et al. [47] successfully introduced the idea of annealing into the field of combinatorial optimization in 1983 and developed a stochastic method for solving a large-scale combinatorial optimization problem. The proposed stochastic method is based on the similarity between the solution procedure of the optimization problem and the annealing process of the physical system and appropriately controls the temperature drop process to achieve simulated annealing by using the Metropolis algorithm so as to achieve the goal of solving the global optimization problem.

Consequently, the fundamental principles and benefits of the aforementioned algorithms are combined with this paper. The natural number coding method is transformed into the route taken by the medical waste recycling vehicle, the C–W algorithm rules construct the initial solution, two distinct fitness functions are set for iterative search, and multiple retention strategies are defined by combining the simulated annealing algorithm, further widening the search space. In addition, the disturbance mechanism is improved to guarantee that the algorithm can jump out of the optimal local solution and enhance the search capability of the solution space to obtain the final optimal route.

### 3.1. Solution Representation

The natural number coding method is introduced: ‘0’ represents the warehouse, and ‘1, 2, 3, …, i, … n ’ indicate the different medical institutions. Meanwhile, to facilitate the design of the neighborhood search operator, the neighborhood solution consists of the execution sequence of each medical waste recycling vehicle. For example, 9 points requiring three medical waste recycling vehicles to provide the service are coded as 03720, 045190, 0680, which means the first medical waste recycling vehicle departs the recycling and processing center and returns to the center after points 3, 7, 2, called sub-route 1; the second vehicle also departs the recycling and processing center and returns to the center after points 4, 5, 1, 9, called sub-route 2; the third vehicle departs the recycling and processing center and returns to the center after points 6, 8, called sub-route 3. The corresponding recycling routing is shown below.

Sub-route 1: 0-3-7-2-0
Sub-route 2: 0-4-5-1-9-0
Sub-route 3: 0-6-8-0

### 3.2. Initial Solution Construction

The initial solution is constructed through the C–W algorithm, and a hard time window constraint is added to the traditional C–W algorithm, i.e., as many points as possible are inserted into a vehicle under the condition that all the constraints are satisfied. Although the solution accuracy is not as good as the ant colony algorithm or the genetic algorithm, a near-optimal satisfactory solution can be obtained quickly. The specific steps are as follows:

Step 1: Estimate the frequency of points and construct the odograph. The vehicle route needs to be arranged for a day if the cycle is A. Construct some empty sets of cycles, and then all nodes are classified according to service frequency and randomly added to different sets. The shortest distance from the depot to the customer node and between nodes is listed.

Step 2: Generate feasible solutions. According to the shortest route calculated from step 1, the medical waste recycling vehicle departs the vehicle parking point to each point one by one, and only one medical waste recycling vehicle is assigned to a point of service under the constraints, such as the rated load capacity of the medical waste recycling vehicle and the time window. Eventually, it sums up the total number of vehicles used to complete this recycling and the total mileage traveled for the recycling.

**Figure 2:**
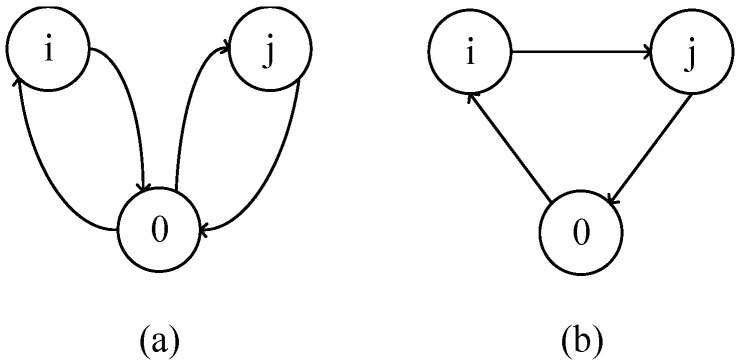
The basic logic of the C–W algorithm. (**a**) The two nodes are transported separately. (**b**) The two nodes are combined into a loop for transport.

Step 3: Calculate the mileage savings between each point. According to the fundamental principle of the C–W algorithm:$S(i,j)=C_{oi}+C_{io}+C_{oj}+C_{jo}-(C_{oi}+C_{ij}+C_{jp})=C_{oi}+C_{oj}-C_{ij}$; that is, to calculate the distance required for a vehicle to start from 0 point, visit node *i* and *j* separately, and then return to 0 point; besides the mileage required for the vehicle to start from 0 point, visit node *i* and *j* in turn, and then return to 0 point. The mileage saved is expressed as the mileage between any two nodes *i* and *j* completed by the same delivery vehicle during the visit process minus the shortest distance between any two nodes *i* and *j*. The aforementioned logic is shown in Figure 2.

Step 4: Rank the mileage savings. The number of miles that can be saved from every point is obtained according to the mileage savings calculated in step 3. Arrange the value of the saved mileage corresponding to the route connected between each two nodes in descending order considering nodes *i* and *j* corresponding to the element max *S(i,j)* with the largest value in the mileage saving table.

Step 5: Merge the recycling routing loops. Rank the mileage saving values according to the mileage saving ranking table; the point with the largest mileage saving is connected in priority, and the loop is merged if two points can use the same medical waste recycling vehicle under the constraints of load capacity and time window. That is, disconnect the arcs between points *i*, *j,* and the vehicle parking point and link the arcs between *i* and *j* to obtain a new loop (o, …, *i, j*, …, o). Repeat this process until there is no more loop that can be merged.

Step 6: Determine the optimal solution. Keep repeating step 5 and comparing each solution until no better solution emerges.

### 3.3. Neighborhood Search Operators

Eight novel operation operators are designed to extend the diversity of neighborhood solutions: the shift operator, the cross operator, the relocate operator, the inter-exchange operator, the intra-exchange operator, the λ-inter-exchange operator, the λ-intra- exchange operator, and the k-opt operator. Besides, the combination of insertion and exchange is utilized to enlarge the solution space. The specific meanings and operation processes of these operators are as follows:

- *Shift* operator: removes a point from its current position and inserts another position in the same route.
- *Cross* operator: given to points on the different routes, exchange the position of the first point and its successor in the same route with the second point and its successor in a different route.
- *Relocate* operator: removes a point from its current position and inserts it into another position with a different route.
- *Inter-exchange* operator: takes two points on different routes and swaps them.
- *Intra-exchange* operator: takes two points from the same route and swaps them.
- The *λ-inter-exchange* operator: select a sub-chain with arbitrary length *λ*_1_ from one route and select another sub-chain with random length *λ*_2_ from a different route, and then swap the two selected sub-chains.
- The *λ-intro-exchange* operator: is similar to the previous operator, and the only difference is the operation carries out on the same route.
- The *k*-*opt* operator: generate a neighboring solution by using *2-opt* at first. If no better solution can be found by *2-opt*, a new neighbor solution will be generated by *3-opt*. When *3-opt* can produce a better solution, the operator returns to *2-opt*. Otherwise, the operator will seek *4-opt*. Repeat the above process until achieving *k-op*t.

**Figure 3:**
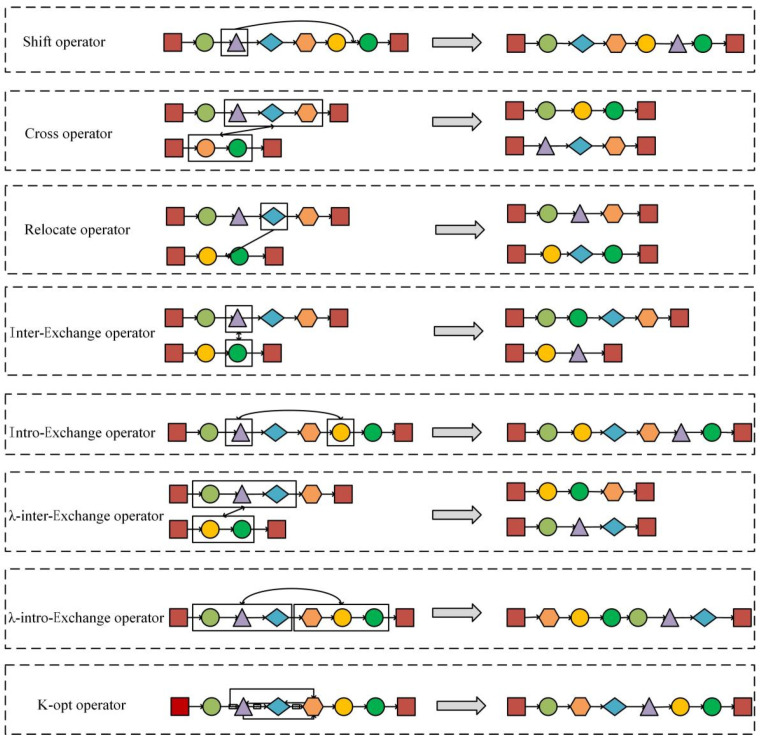
The operation processes of neighborhood search operators.

The operation processes of the above operators are shown in Figure 3.

### 3.4. Structural Rules of the Solution

**Figure 4:**
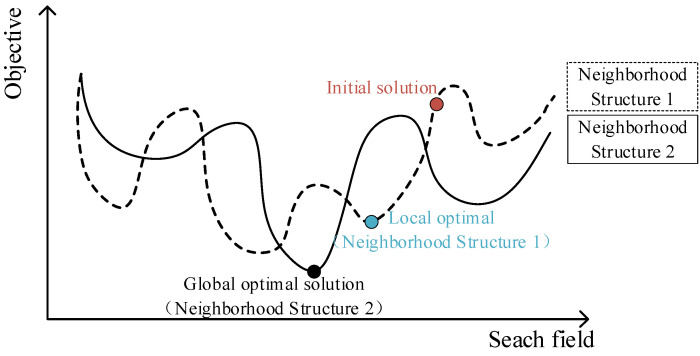
Illustration of the local search process.

The traditional variable neighborhood search algorithm has the following characteristics when solving combinatorial optimization problems, as shown in Figure 4: (1) the local optimal solutions may be different when the alternate search is carried out by using the neighborhood structures composed of different actions in the same search area. (2) The optimal local solution obtained for one neighborhood structure is not necessarily the optimal local solution for another neighborhood structure, and the algorithm is easy to fall into the local optimum and stop. (3) The final global optimal solution must be an optimal local solution of a neighborhood structure. Therefore, the basic idea of the simulated annealing algorithm is further integrated into the improved variable neighborhood search algorithm so as to increase the diversity of solutions and jump out of the local optimum. When the fitness value of the left or right neighborhood is smaller than its own fitness, an attempt is made to accept it for the initial value of the next iteration with a certain probability; i.e., the existence of infeasible solutions is temporarily allowed. There are two types of infeasible solutions in the neighborhood search: (1) the actual load of the medical waste recycling vehicle in the solution sequence exceeds the maximum load capacity; (2) the arrival time of a point in the solution sequence violates its time window range. In addition, we list the case where the number of vehicles in the feasible solution is reduced as the first-level indicator, and the case where the number of vehicles is not reduced but the total distance is shortened is listed as the second-level indicator. Similarly, the reduction in the number of vehicles in the infeasible solution is listed as the first-level index, and the situation where the number of vehicles is not reduced but the total distance is shortened is listed as the second-level index [48]. Consequently, the corresponding penalty function is added to the objective function during the search process to deal with the infeasible solution, and then a new fitness function *f* is formed as the following equation:

(14) $$f=\sum_{v\in V}\sum_{i\in N}\sum_{j\in N}\sum_{t\in T}c_{ij}•x_{ijv}^{t}+\lambda_{1}\sum_{v\in V}\sum_{i\in N}\max(0,s_{i}^{v}-e_{i})+\lambda_{2}\sum_{v\in V}\sum_{i\in N}\sum_{t\in T}\max(0,q_{i}^{t}-Q)$$

The right-hand side of the above equation is the transportation cost of the route, the penalty function for violating the time window, and the penalty function for violating the load capacity, where *λ*_1_ and *λ*_2_ are the penalty coefficients of the time window constraint and the load capacity constraint, respectively, with an initial value of 0.5. When the constraint of time and cargo capacity constraint are satisfied, the penalty coefficient becomes smaller, which is *λ*_1_/(1 + *λ*_1_),*λ_2_*/(1 + *λ*_2_), respectively, and True is returned. When the constraint of the time window or the cargo capacity constraint is violated, the corresponding penalty coefficient becomes larger, which is *λ*_1_ × (1 + *λ*_1_), *λ*_2_ × (1 + *λ*_2_), respectively, and returns False.

### 3.5. Perturbation Mechanisms

**Figure 5:**
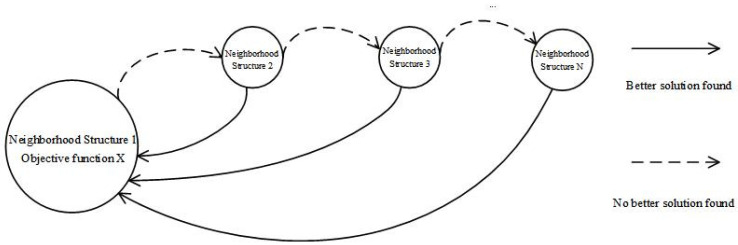
Illustration of the perturbation process.

The main purpose of the perturbation process is to obtain a large number of random solutions through some transformation. Each neighborhood structure needs to find a balance between sufficiently perturbing the current solution and retaining the better-quality part of the current solution, thus expanding the search space of the current solution and avoiding falling into a local optimum. As shown in Figure 5, the perturbation frequency of the traditional variable neighborhood search algorithm is too high, so the algorithm cannot thoroughly search the current neighborhood solution and jump into another neighborhood structure, and the objective function changes accordingly. Because the randomly generated neighborhood solution deviates significantly from the original neighborhood solution after perturbation, the search for the current neighborhood solution is not comprehensive enough. Therefore, the target value of the currently generated neighborhood solution is set to infinity by the improved variable neighborhood search algorithm when no optimal solution is found after Y consecutive iterations of the current neighborhood structure, and the newly generated neighborhood solution will replace the neighborhood solution with the infinite target value and be passed down during the next traversal of the operation operator. Eventually, an optimal local solution with good quality will be obtained again while speeding up the algorithm by continuously seeking and replacing different neighborhood structures.

### 3.6. IVNS Procedure

**Figure 6:**
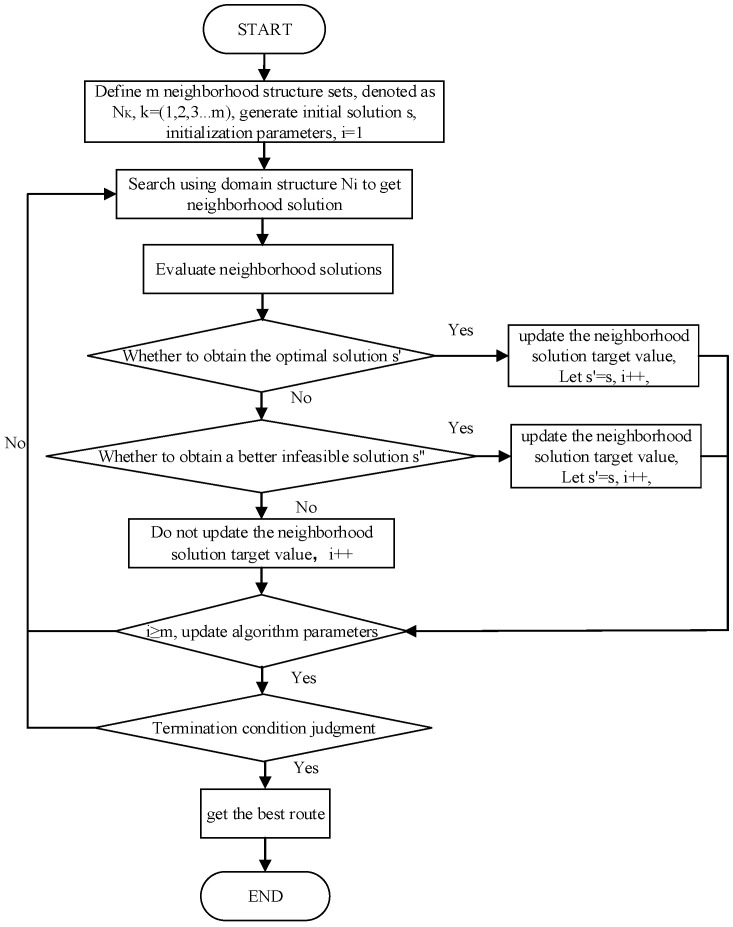
Algorithm flow chart.

The specific procedure of the proposed IVNS algorithm is shown in Figure 6. First, a feasible initial solution is generated by the C–W algorithm, the set of neighborhood structures is defined, the parameters are initialized, and then the main algorithm is passed in. Second, each newly generated neighborhood solution is calculated and evaluated during the neighborhood search: the target value is updated and passed to the global optimum if the optimized solution is obtained; the target value is also updated if no optimized solution is obtained and a better infeasible solution is searched; if neither of the above occurs, there is no updated solution obtained in this iteration. Finally, the termination condition is determined, and the algorithm is terminated if the number of iterations reaches a given threshold.

## 4. Computational Experiment and Case Study

In this section, 20 PVRPTW are the benchmarks published by Cordeau et al. [47], and actual cases of medical waste recycling in some tertiary medical institutions in Beijing were adopted to evaluate the performance of the IVNS. Meanwhile, the commercial optimization software Gurobi 9.5.0 was used for comparative experiments. For the small-scale case, the maximum solution time of Gurobi was set to be 1800 s to output the current optimal feasible solution, while, with regard to the large-scale case, the maximum iteration time was set to be 3600 s because a feasible solution cannot be obtained in a short time. Both the proposed INVS and Gurobi were implemented in Python and accomplished in the language editor Anaconda on a PC with AMD Ryzen 5 4600U with Radeon Graphics 2.10 GHz and 8 GB of RAM.

### 4.1. Parameter Settings

Different parameter settings will have a direct impact on the performance of the algorithm. Three dominant parameters of the proposed IVNS algorithm are: the maximum number of iterations M, the maximum number of continuous iteration results unchanged B, and the frequency of disturbance Y. Based on the preliminary test, the maximum number of iterations M is set to 1800, and the value ranges of the other two parameters that need to be adjusted are as follows:

B: 100, 200, 300, 400.
Y: 5, 10, 15, 20.

**Figure 7:**
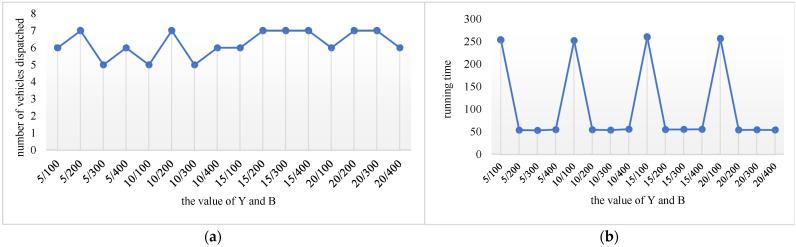
The experimental results of parameters. (**a**) The number of vehicles with different values of B, Y. (**b**) Running time with different values of B, Y.

The C101 example in the Solomon instance that contains 100 customers is utilized for the parameter testing. The test results show that the number of dispatched vehicles is the least when the number of iterations is 500, Y = 5, B = 300 or Y = 10, B = 100 or Y = 10, B = 300. When the number of iterations is 1800, Y = 10, B = 300, the running time is the shortest. Finally, Y is set to be 10, and the value of B is 300 (Figure 7).

### 4.2. Computational Experiment on Benchmarks

#### 4.2.1. Dataset

The 20 PVRPTW benchmarks are shown in Table 2, where the difference between the first ten and the last ten cases lies in the time window. Take the Pr01 case with a cycle of 4 as an example, and the parameters are presented in Table 3. The frequency of recycling represents the number of times that a point is recycled during the cycle. Each visit combination is coded with the decimal equivalent of the corresponding binary bit string. For example, in a 5-day period, the code 10, which is equivalent to the bit string 01010, means that a customer is visited on days 2 and 4 (days are numbered from left to right).

#### 4.2.2. Results and Analysis

The computational results of benchmarks are shown in Table 4, where the second column indicates the known optimal solution published on the website [49]; the third column is the best computational results on 10 test questions with improved ant colony optimization (IACO) [50]; the fourth column represents the initial solution obtained by the C–W algorithm; the fifth to seventh columns demonstrate the maximum values, average values, and optimal values obtained by running the IVNS algorithm ten times for each example separately. The eighth column shows the results solved by the Gurobi optimizer. The ninth to eleventh columns represent the relative percentage error between the optimal value obtained by the IVNS algorithm and the optimal solution of the other algorithms. The twelfth column presents the optimal number of tours on a certain day. The last column shows the running time of the instances. The error is calculated as follows [51]:

(15) $$RPD=\frac{S\mathrm{olution}-BKS}{BKS}\times 100\%$$

where the solution is the optimal result obtained by the improved variable neighborhood search algorithm, and BKS is the known optimal solution. It can be seen from Table 4 that the average value of the worst solutions in the 10 results of the improved variable neighborhood search algorithm is 8966.67, the average value of the optimal solutions is 8609.09, and the deviation between the mean solution and the worst solution after 10 runs is 1.85%, and the deviation between the mean solution and the optimal solution is −2.25%. It can thus be seen that the proposed IVNS algorithm can converge to the optimal solution stably for PVRPTW. Furthermore, in terms of RPD metrics, three optimal solutions acquired by the improved variable neighborhood search algorithm are better than the known optimal solutions published on the website, and the difference between the mean value of the 20 cases and the average optimal solutions is 1.9%. Compared with an improved ant colony optimization algorithm, the difference between the mean value of the first ten cases and the average optimal solutions is 0.89%. Both improved algorithms are obviously superior to the Gurobi optimizer in terms of solution quality. Overall, the improved variable neighborhood search algorithm can obtain better results under any data size, while the Gurobi optimizer cannot generate a feasible solution when the number of points exceeds 200. Consequently, the proposed IVNS has strong effectiveness and feasibility in solving PVRPTW.

**Figure 8:**
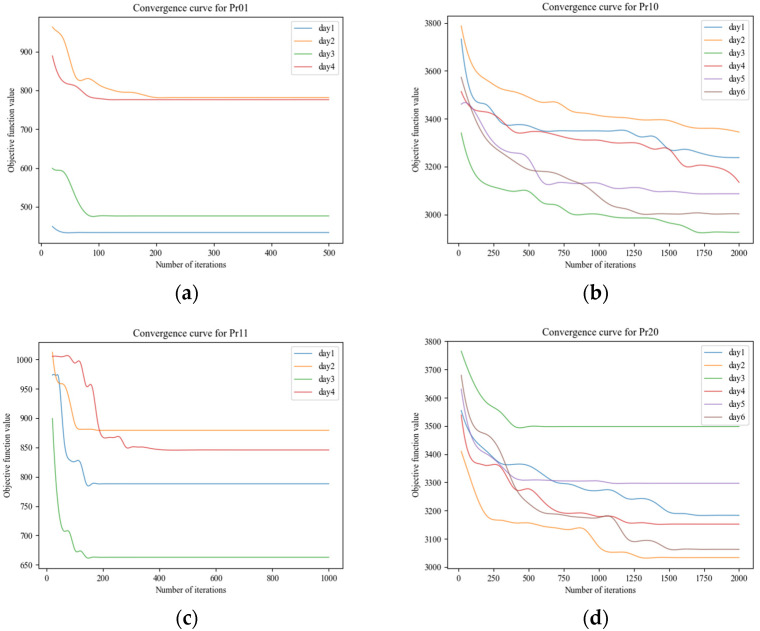
Convergence curves of IVNS for four instances. (**a**) Convergence curve for Pr01. (**b**) Convergence curve for Pr10. (**c**) Convergence curve for Pr11. (**d**) Convergence curve for Pr20.

To further test the performance of the proposed IVNS, the convergence validation was implemented through computational experiments. We run the IVNS algorithm with the instances Pr1 (48 pints, 4 days in a cycle, narrow time window), Pr10 (288 points, 6 days in a cycle, narrow time window), Pr11 (48 points, 4 days in a cycle, wide time window), and Pr20 (288 points, 6 days in a cycle, wide time window), respectively. The convergence curves of the instances are shown in Figure 8. It can be seen in Figure 8 that the target value of instance Pr01 stabilized at around 3000 when the number of iterations reached 150, while the target value of example Pr10 is around 17,800 after 2000 iterations. It shows the IVNS can obtain the optimal global solution in a few iterations for the small size problem. For the large size problem, the value of the objective function decreased greatly in the early stage, and the decline gradually slowed down as the iterations increased. In general, the improved variable neighborhood search algorithm is found to be capable of converting to an optimal value with a fast convergence speed; thus, the superiority of the algorithm is verified.

### 4.3. Case Study

#### 4.3.1. Dataset and Parameter Setting

The case study of medical waste recycling was conducted by using real data from 108 tertiary medical institutions in which the amount of medical waste generated accounts for 56.7% of the medical waste generated by all the medical institutions in Beijing. The medical waste recycling is carried out by Beijing Sanitation Group Operation Co., Ltd., which has a total of *K* = 5 dedicated medical waste recycling vehicles with a maximum load capacity of *Q* = 7 tons and a speed of *s* = 40 km/h. The medical waste recycling and processing center is Gaoantun; the daily waste production and recycling time of the selected 108 medical waste recycling points without time window are attached in Appendix A. Meanwhile, the daily waste production, recycling time, and time window of the selected 31 medical waste recycling points are presented in Table 5. According to the Chinese medical waste recycling policy, the recycling frequency is once every two days when the daily waste production is less than 0.3 tons. Besides, in accordance with the actual traffic and control situation in Beijing, the medical waste recycling work starts as early as 5 a.m. and ends as late as 8 p.m., with a maximum vehicle working time of *T*max = 10 h. The medical waste loading time per ton is 0.5 h, the fixed cost of each vehicle departure is CNY 400, and the unit transportation cost of the vehicle is CNY 8 per kilometer.

#### 4.3.2. Results and Analysis

The proposed model and IVNS algorithm are adopted for calculation, setting the number of iterations to 2000. The optimal route for recycling of 31 nodes is shown in Table 6, where the second column indicates the number of vehicles to be used under the cyclical recycling strategy, the third column expresses the number of vehicles to be used without considering the cyclical condition, and the fourth column represents the docking sequence of each medical waste recycling vehicle at medical institution points under the cyclical recycling strategy. The fifth column shows the docking sequence of each medical waste recycling vehicle at medical institution points without considering the cyclical recycling strategy. For example, 0-3-1-4-20-0 means the medical waste recycling vehicle departs from the recycling and processing center and successively serves medical institution points 3, 1, 4, 20, and finally returns to the recycling and processing center.

**Figure 9:**
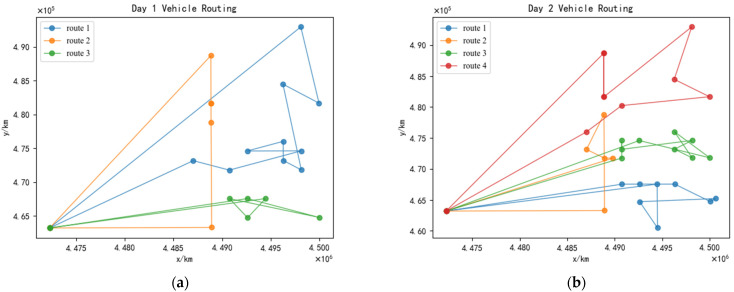
The optimal recycling schemes under the cyclical recycling strategy. (**a**) Medical waste recycling vehicle route of the first day. (**b**) Medical waste recycling vehicle route of the second day.

**Figure 10:**
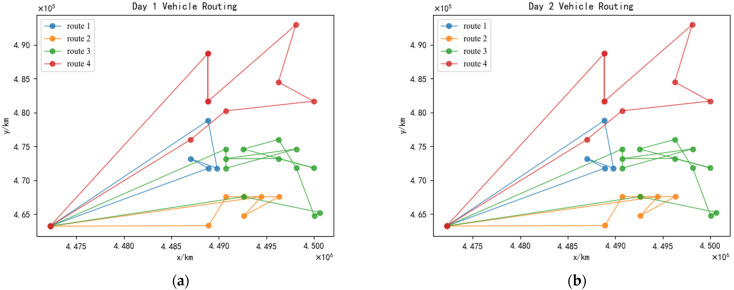
The optimal recycling schemes without considering the cyclical condition. (**a**) Medical waste recycling vehicle route of the first day. (**b**) Medical waste recycling vehicle route of the second day.

It can be observed from Table 6 that three medical waste recycling vehicles are required on the first day and four vehicles on the second day under the cyclical recycling strategy. The recycling loads of each medical waste recycling vehicle on the first day are 6.9912 t, 3.0504 t, and 4.1632 t, respectively; the recycling working times of each medical waste recycling vehicle are 11.6 h, 7 h, and 10 h; and the distances traveled by the medical waste recycling vehicles are 80.198 km, 52.361 km, and 60.00 km. Meanwhile, on the second day, the recycling loads of each medical waste recycling vehicle are 4.3152 t, 3.1736 t, 4.96 t, and 4.3216 t, respectively; the recycling working times of each medical waste recycling vehicle are 10 h, 7 h, 8.23 h, and 7.92 h; and the distances traveled by the medical waste recycling vehicles are 60 km, 34.142 km, 44.721 km, and 74.787 km. Comparatively, there are four vehicles needed for recycling on both days without considering the cyclic condition. The recycling loads of each medical waste recycling vehicle are 2.2176 t, 3.4968 t, 6.581 t, and 3.8432 t, respectively. Further, the recycling working times of each medical waste recycling vehicle are 6.41 h, 8 h, 9.41 h, and 7.82 h, and the distances traveled by the medical waste recycling vehicles are 28.284 km, 40 km, 66.50 km, and 74.78 km. According to the above results, we can find that the total number of vehicles dispatched, recycling loads, recycling time, and the distance traveled by the vehicle under the medical waste recycling vehicle routing planning schemes, considering the cyclical recycling strategy, are smaller than the medical waste recycling vehicle routing planning schemes without considering the cyclical condition. Moreover, the optimal recycling schemes under the cyclical recycling strategy and the optimal recycling schemes without considering the cyclical condition are shown in Figure 9 and Figure 10, respectively. There are differences in the number of medical waste recycling vehicles used and the vehicle route under the two schemes for the same medical institution point during the practical recycling process: the number of vehicles used and the recycling routes under the consideration of cyclical conditions are more scientific, thereby making the recycling more efficient.

In addition, an observation of the results in Table 7 for the instance of 31 nodes is that four medical waste recycling vehicles are required on both days. The total cost is 7236 RMB under the constraints of vehicle load capacity and time window without considering the cyclical condition, while, for the same medical institution point and constraints, only three medical waste recycling vehicles are required on the first day. Four medical waste recycling vehicles are required on the second day, with a total cost of 6419.2 RMB under the cyclical recycling strategy, which is 12.7% lower than the total cost without considering the cyclical condition. For the instance of 108 nodes, a total of 40 vehicles are needed in two days, and the cost is 29872 RMB without considering the cyclical condition. Further, considering the cyclical recycling strategy, a total of 37 vehicles are needed in two days with a total cost of 26176 RMB, which is 12.3% lower than the total cost without considering the cyclical condition. Consequently, the model that considers the cyclical recycling strategy has stronger superiority for the medical waste recycling vehicle routing problem.

## 5. Conclusions and Remarks

### 5.1. Conclusions

Efficient recycling of urban medical waste is of great importance for protecting public health and reducing environmental pollution. It is regarded as a cyclical medical waste recycling vehicle routing optimization problem with time windows on the basis of analyzing the network structure of medical waste recycling and fully considering the timeliness of medical waste recycling. To study the multi-cycle medical waste recycling vehicle routing problem with time windows, we conducted a comprehensive literature review on medical waste recycling, the vehicle routing problem with time windows, the periodic vehicle routing problem, and metaheuristics for these problems. Meanwhile, a mixed-integer programming model was established to minimize the number of dispatched vehicles and transportation costs. At the same time, in order to enhance the search ability of the algorithm to the solution space, an improved variable neighborhood search algorithm was proposed to optimize the construction of the initial solution and the disturbance mechanism. With the purpose of verifying the proposed algorithm, the comparison experiment with the benchmarks of PVRPTW was carried out. Eventually, a case study of the medical waste recycling was conducted by utilizing the real data from 108 tertiary medical institutions in Beijing. The computational results show that the optimal value obtained by the proposed model and algorithm can afford a more scientific and efficient medical waste recycling vehicle route so as to achieve the goal of reducing transportation time and cost. 

To sum up, we can draw the following conclusions:

(1) The mixed-integer programming model is an effective approach that can accurately reflect the characteristics of the multi-cycle medical waste recycling vehicle routing optimization problem with time windows.
(2) For the parameter settings of IVNS, the performance of the algorithm is best when the maximum number of iterations M is 1800, the maximum number of continuous iterations results unchanged B is 300, and the frequency of disturbance Y is 10.
(3) The improved variable neighborhood search algorithm (IVNS) performs better than the improved ant colony optimization (IACO) and C–W algorithm. This is mainly because the IVNS can work out the slow convergence rate and premature problem through a novel initial solution generation scheme and a better solution search mechanism.
(4) The total number of vehicles dispatched, recycling loads, recycling time, and distance traveled by the vehicle under the periodic strategy are smaller than those of the single-cycle strategy because the periodic scheme can achieve the maximization of the vehicle’s utility.

### 5.2. Future Scope of Research

The presented decision-making framework provides a novel procedure for the medical waste recycling vehicle routing optimization. Although both the model and the approach are innovative, there are some extensions suggested for future works. First, the multiple objectives that especially involve service quality will be considered, and the characteristics of medical waste, such as category and infectivity, will be further integrated into the model. Second, the performance of IVNS will be tested in the multi-objective condition. Third, the proposed model does not take the fuzzy demand into account. The uncertain demand could have an important impact on the medical waste recycling vehicle routing problem. Owing to the consideration of the NP-hardness of the proposed model, the involvement of uncertain demand is limited, but it would be a useful issue for further study.

### 5.3. Managerial Implications

In this paper, we propose a decision-making approach for the multi-cycle medical waste recycling vehicle routing problem with time windows with respect to demand fulfillment and cost saving.

(1) Based on the real-time data of the municipal medical waste recycling network, managers can dispatch the medical waste recycling vehicles optimally according to the scheme provided by the proposed approach.
(2) Considering the resources, that is, the fixed components (e.g., medical waste recycling and processing center) and flexible components (e.g., medical waste recycling vehicles and workers), the enterprises can increase or decrease the flexible components on the basis of the actual situations so as to minimize the operating cost.
(3) Metaheuristics are the essential point of artificial intelligence technology, which is developing rapidly at present. Considering that our proposed approach is metaheuristic-based, not only the researchers but also the enterprises can use it conveniently through Python, MATLAB, or other tools.

## Figures and Tables

**Table 1:** Notations of the mathematical model.

**Sets**	
**T**	set of cycle length, T = {1, 2,3, …, *t*} days
**N**	The set of medical institutions, N = {1, 2, 3… *n*}, 0 and *n* + 1 are vehicle parking points, *N*_0_ = N∪{0, *n* + 1}
**V**	Set of vehicles, *V* = {1,2,3 … *v*}
**Parameters**	
*c_ij_*	Distance from medical institution *i* to medical institution *j*, *i, j∈N*_0_
*t_ij_*	Transportation time from medical institution *i* to medical institution *j*, *i, j∈N*_0_
*se_i_*	Service time for medical institution *i*, *i* *∈ N*_0_, where *se*_0_ *= se_n+_*_1_ *= 0*
*e_i_*	The lower bound of time window for medical institution *i*
*l_i_*	The upper bound of time window for medical institution *i*
*ω*_1_	Unit fixed usage costs of vehicle
*ω*_2_	Unit travel cost of vehicle
*Ψ*	Maximum value
*Q*	Maximum load capacity of medical waste recycling vehicles
$q_{i}$	Amount of medical waste generated by medical institution *i* in a cycle
**Decision-making variables**	
$f_{i}$	The required recycling frequency of medical institution *i* in a cycle (The amount of waste that needs to be recycled each day within a cycle of medical institution *i* is *q_i_/f_i_*)
$x_{ijv}^{t}$	Binary variables: 1 if vehicle *v* arrives directly at medical institution *j* from medical institution *i* on day *t*; 0 otherwise
$y_{iv}^{t}$	Binary variables: 1 if vehicle *v* visits medical institution *i* on day *t*; 0 otherwise
$s_{i}^{v}$	Arrival time of vehicle *v* at medical institution *i*

**Table 2:** The parameters of PVRPTW benchmarks.

No.	Number of Points	Number of Days in a Cycle	Number of Vehicles	Maximum Duration of a Route	Load Capacity
Pr01	48	4	3	500	200
Pr02	96	4	6	480	195
Pr03	144	4	9	460	190
Pr04	192	4	12	440	185
Pr05	240	4	15	420	180
Pr06	288	4	18	400	175
Pr07	72	6	4	500	200
Pr08	144	6	8	475	190
Pr09	216	6	12	450	180
Pr10	288	6	16	425	170
Pr11	48	4	3	500	200
Pr12	96	4	6	480	195
Pr13	144	4	9	460	190
Pr14	192	4	12	440	185
Pr15	240	4	15	420	180
Pr16	288	4	18	400	175
Pr17	72	6	4	500	200
Pr18	144	6	8	475	190
Pr19	216	6	12	450	180
Pr20	288	6	16	425	170

**Table 3:** The parameters of case Pr01.

Point Number	Horizontal Coordinate	Vertical Coordinate	Service Duration	Demand	Frequency of Recycling	Number of Possible Visit Combinations	List of All Possible Visit Combinations	Beginning of Time Window	End of Time Window
0	−10.442	19.999	0	0	0	0		0	1000
1	−29.730	64.136	2	12	4	1	15	354	509
					…				
13	−76.672	99.341	2	9	2	2	5 10	368	528
					…				
48	42.883	−2.966	17	10	1	4	1 2 4 8	98	233

**Table 4:** Results of PVRPTW benchmarks.

No.	The Vrp Web	IACO	Initial Solutions	IVNS			Gurobi	RPD			The Number of Tours in Each Day	Average Time
				Max	AVG	Best		The Vrp Web	IACO	Gurobi		
Pr01	3007.84	2959.09	4124.74	3275.52	3190.55	3048.54	5326	1.35%	3.02%	77.07%	3 + 3 + 3 + 3 = 12	28.75
Pr02	5328.33	5323.29	5870.59	5786.76	5544.62	5421.99	9972.2	1.75%	1.85%	87.15%	6 + 5 + 5 + 6 = 22	225.75
Pr03	7397.10	7554.5	10,289.47	7991.69	7747.29	7578.25	17,747.6	3.1%	0.31%	139.93%	7 + 7 + 8 + 7 = 29	770.52
Pr04	8376.95	8364.61	10,438.8	8724.81	8694.54	8589.03	29,599	2.53%	2.68%	253.34%	10 + 8 + 9 + 8 = 35	1364.56
Pr05	8967.90	8964.46	11,132.58	9332.93	9195.34	9008.26	-	0.45%	0.48%	-	10 + 12 + 10 + 11 = 43	2828.25
Pr06	11,686.91	11,122.6	14,027.47	11,541.02	11,407.5	11,095.82	-	-5.05%	-0.24%	-	12 + 12 + 12 + 12 = 48	5148.25
Pr07	6991.54	7100.24	9528.99	7587.05	7339.47	7188.22	12,458.8	2.81%	1.23%	78.20%	5 + 5 + 5 + 4 + 5 + 4 = 28	117.66
Pr08	10,045.05	10,094.58	13,321.33	11,081.46	10,957.89	10,469.85	26,324.2	4.22%	3.71%	162.06%	7 + 7 + 7 + 8 + 7 + 7 = 43	602.51
Pr09	14,294.97	14,356.9	18,760.46	14,757.65	14,562.75	14,261.85	-	-0.23%	-0.66%	-	10 + 10 + 9 + 10 + 10 + 10 = 59	2239.59
Pr10	18,609.72	17,733.2	22,274.61	18,572.11	18,228.6	17,751.03	-	-4.61%	1.00%	-	12 + 12 + 14 + 11 + 13 + 12 = 74	4950.83
Pr11	2318.37	-	3169.73	2612.77	2502.82	2449.15	5470.3	5.74%	-	135.95%	3 + 2 + 3 + 2 = 10	28.75
Pr12	4276.13	-	4765.68	4765.68	4718.11	4582.77	10,295.4	7.17%	-	140.76%	5 + 5 + 5 + 4 + 6 + 5 = 30	189.25
Pr13	5702.07	-	7847.23	6676.34	6510.41	6315.38	20,775.4	10.75%	-	264.35%	5 + 7 + 6 + 6 = 24	551.51
Pr14	6789.73	-	8577.73	7373.38	7224.04	7053.58	20,890.2	3.88%	-	207.67%	7 + 8 + 7 + 8 = 30	1760.23
Pr15	7102.36	-	8966.93	7705.69	7633.47	7554.06	-	6.66%	-	-	9 + 11 + 10 + 10 = 40	2205.64
Pr16	9180.15	-	11,984.49	9762.46	9559.34	9348.02	-	1.82%	-	-	11 + 11 + 11 + 11 = 44	4304.91
Pr17	5606.08	-	6097.75	6097.75	5999.52	5876.42	13,371.4	4.82%	-	138.52%	4 + 4 + 4 + 3 + 5 + 4 = 24	74.66
Pr18	7987.64	-	11,246.73	8635.34	8574.79	8426.2	25,550.2	5.83%	-	219.87%	7 + 6 + 6 + 6 + 7 + 6 = 38	587.78
Pr19	11,089.91	-	14,849.32	12,085.83	11,812.88	11,515.86	-	5.57%	-	-	8 + 9 + 8 + 8 + 8 + 9 = 50	2167.38
Pr20	14,207.64	-	14,687.4	14,967.31	14,668.88	14,355.56	-	1.04%	-	-	12 + 12 + 12 + 12 + 12 + 12 = 72	4112.16
Average	8448.32	-	10,598.1	8966.67	8803.64	8609.09	-	1.9%	-	-	-	1712.95

**Table 5:** The coordinates, loading time, daily waste production, and time window of the selected points.

No.	Horizontal Coordinate	Vertical Coordinate	Loading Time (h)	Medical Waste Volume (t/d)	Start time (h)	End Time (h)
0	4,472,263	463,203	0	0	5	20
1	4,487,027	473,163	0.574	1.148	7	9
2	4,490,734	471,765	0.3608	0.7216	7.5	9
3	4,488,883	471,758	0.1208	0.2416	8	10
4	4,489,808	471,761	0.014	0.028	9	10.5
5	4,492,574	474,594	0.344	0.688	11.5	13.5
6	4,490,729	473,176	0.0616	0.1232	9.5	11.5
7	4,496,281	473,196	0.1608	0.3216	13	14.5
8	4,488,920	463,285	0.3432	0.6864	12.5	14
9	4,492,615	464,714	0.4	0.8	10	12
10	4,490,751	467,545	0.56	1.12	13.5	16
11	4,492,602	467,553	0.4	0.8	15.5	17.5
12	4,494,453	467,553	0.2424	0.4848	7	8.5
13	4,490,751	474,613	0.122	0.244	8.5	10.5
14	4,496,303	467,553	0.14	0.28	11.5	14
15	4,498,127	474,613	0.4528	0.9056	10	12.5
16	4,496,271	476,018	0.1836	0.3672	13	16
17	4,496,249	484,482	0.24	0.48	14	16.5
18	4,488,838	488,703	0.322	0.644	9	11
19	4,488,852	481,642	0.204	0.408	9.5	12.5
20	4,488,860	478,818	0.4	0.8	13	15
21	4,498,137	471,793	0.5192	1.0384	12.5	15
22	4,487,017	475,988	0.12	0.24	15.5	17.5
23	4,488,838	488,703	0.1072	0.2144	7	9
24	4,490,707	480,235	0.012	0.024	12	14.5
25	4,499,957	481,670	0.2604	0.5208	14	15.5
26	4,499,988	471,800	0.1208	0.2416	8.5	15.5
27	4,488,852	481,642	0.256	0.512	8	14.5
28	4,498,087	492,948	0.4	0.8	10.5	17.5
29	4,500,018	464,749	0.4792	0.9584	11.5	16
30	4,500,633	465,222	0.0852	0.1704	8	15.5
31	4,494,487	460,490	0.0628	0.1256	9	12

**Table 6:** Optimal route for medical waste recycling vehicles with and without considering the cyclical recycling strategy.

Number of Days	Route Number		Order of Stopping at Medical Institution Points	
	A	B	A	B
1	1	1	0-1-2-15-5-16-7-21-17-25-28-0	0-3-1-4-20-0
	2	2	0-18-19-27-20-8-0	0-12-9-14-31-10-8-0
	3	3	0-12-9-10-29-11-0	0-13-2-15-6-7-26-5-16-21-29-30-11-0
		4		0-18-19-23-27-28-17-25-24-22-0
2	1	1	0-12-31-9-30-29-14-11-10-0	0-3-1-4-20-0
	2	2	0-4-3-1-20-8-0	0-12-9-14-31-10-8-0
	3	3	0-2-13-6-15-7-21-16-26-5-0	0-13-2-15-6-7-26-5-16-21-29-30-11-0
	4	4	0-18-19-23-27-28-17-25-24-22-0	0-18-19-23-27-28-17-25-24-22-0

**Table 7:** The comparison of number of vehicles and total cost under two schemes.

	Number of Days	Instance with 31 Nodes		Instance with 108 Nodes	
		Number of Vehicles	Cost (RMB)	Number of Vehicles	Cost (RMB)
Without cyclical recycling strategy	1	4	3618	20	14,936
	2	4	3618	20	14,936
With cyclical recycling strategy	1	3	2801.2	15	11,072
	2	4	3618	22	15,104

## Data Availability

The data in the research are publicly available. The data can be found here: http://www.bernabe.dorronsoro.es/vrp/ (accessed on 1 November 2021).

## Appendix A

**Table A1:** The coordinates, loading time, and daily waste production of 109 points.

No.	Horizontal Coordinate	Vertical Coordinate	Loading Time (h)	Medical Waste Volume (t/d)
0	4,472,263	463,203	0	0
1	4,485,176	473,156	0.4988	0.9976
2	4,487,027	473,163	0.574	1.148
3	4,485,176	473,156	0.2736	0.5472
4	4,485,175	473,155	0.2592	0.5184
5	4,481,480	471,730	0.3	0.6
6	4,490,734	471,765	0.3608	0.7216
7	4,488,883	471,758	0.1208	0.2416
8	4,481,480	471,730	0.026	0.052
9	4,489,808	471,761	0.014	0.028
10	4,492,574	474,594	0.344	0.688
11	4,490,729	473,176	0.0616	0.1232
12	4,496,281	473,196	0.1608	0.3216
13	4,487,056	466,101	0.2308	0.4616
14	4,488,920	463,285	0.3432	0.6864
15	4,492,615	464,714	0.4	0.8
16	4,490,751	467,529	0.56	1.12
17	4,485,225	461,854	0.1592	0.3184
18	4,483,342	468,910	0.4152	0.8304
19	4,481,485	470,316	0.3624	0.7248
20	4,492,602	467,537	0.4	0.8
21	4,483,354	466,084	0.3124	0.6248
22	4,487,062	464,688	0.3124	0.6248
23	4,494,453	467,545	0.2424	0.4848
24	4,483,361	464,671	0.2196	0.4392
25	4,490,751	467,529	0.122	0.244
26	4,496,303	467,553	0.14	0.28
27	4,481,503	466,076	0.1472	0.2944
28	4,498,127	474,613	0.4528	0.9056
29	4,479,610	477,378	0.4792	0.9584
30	4,496,271	476,018	0.1836	0.3672
31	4,496,249	484,482	0.24	0.48
32	4,488,838	488,703	0.322	0.644
33	4,435,207	472,978	0.288	0.576
34	4,487,152	449,151	0.1192	0.2384
35	4,483,307	478,802	0.1804	0.3608
36	4,488,852	481,642	0.204	0.408
37	4,431,535	465,850	0.2	0.4
38	4,488,860	478,818	0.4	0.8
39	4,498,137	471,793	0.5192	1.0384
40	4,487,017	475,988	0.12	0.24
41	4,488,838	488,703	0.1072	0.2144
42	4,490,707	480,235	0.012	0.024
43	4,433,317	488,619	0.1488	0.2976
44	4,499,957	481,670	0.2604	0.5208
45	4,499,988	471,800	0.1208	0.2416
46	4,488,852	481,642	0.256	0.512
47	4,498,087	492,948	0.4	0.8
48	4,435,249	463,022	0.0992	0.1984
49	4,500,018	464,749	0.4792	0.9585
50	4,500,633	465,222	0.0852	0.1704
51	4,494,487	460,490	0.0628	0.1256
52	4,487,152	449,151	0.34	0.68
53	4,498,196	459,099	0.28	0.56
54	4,485,240	459,028	0.352	0.704
55	4,488,966	454,812	0.296	0.592
56	4,435,249	463,022	0.28	0.56
57	4,501,914	456,300	0.26	0.52
58	4,498,247	450,637	0.12	0.24
59	4,488,984	451,988	0.128	0.256
60	4,485,282	451,964	0.1528	0.3056
61	4,472,285	458,957	0.26	0.52
62	4,475,945	467,465	0.36	0.72
63	4,481,539	459,008	0.2072	0.4144
64	4,474,094	467,457	0.244	0.488
65	4,481,539	459,008	0.048	0.096
66	4,477,764	475,958	0.12	0.24
67	4,475,908	477,367	0.18	0.36
68	4,479,659	464,653	0.284	0.568
69	4,475,908	474,544	0.37	0.74
70	4,470,535	443,375	0.16	0.32
71	4,492,756	442,132	0.128	0.256
72	4,490,894	443,529	0.3224	0.6448
73	4,492,756	442,132	0.32	0.64
74	4,485,342	443,487	0.26	0.52
75	4,485,311	447,726	0.1	0.2
76	4,492,802	436,486	0.1	0.2
77	4,492,878	428,017	0.32	0.64
78	4,485,131	507,064	0.4168	0.8336
79	4,483,281	507,066	0.1008	0.2016
80	4,488,833	507,061	0.2448	0.4896
81	4,481,429	505,654	0.22	0.44
82	4,448,117	492,901	0.232	0.464
83	4,449,967	521,297	0.34	0.68
84	4,448,144	521,297	0.12	0.24
85	4,453,669	507,094	0.15	0.3
86	4,461,075	490,079	0.08	0.16
87	4,468,497	480,177	0.056	0.112
88	4,413,056	460,062	0.3608	0.7216
89	4,461,079	487,244	0.0928	0.1856
90	4,455,614	461,703	0.1468	0.2936
91	4,461,103	477,322	0.15	0.3
92	4,448,268	451,727	0.68	1.36
93	4,444,496	464,488	0.12	0.24
94	4,448,268	451,727	0.12	0.24
95	4,472,355	447,635	0.2584	0.5168
96	4,470,505	447,622	0.1608	0.3216
97	4,470,515	446,207	0.048	0.096
98	4,446,367	460,236	0.2	0.4
99	4,442,610	473,004	0.36	0.72
100	4,440,816	460,200	0.4276	0.8552
101	4,462,984	468,826	0.18	0.36
102	4,448,162	473,024	0.22	0.44
103	4,442,638	465,900	0.6528	1.3056
104	4,457,386	517,017	0.3	0.6
105	4,457,417	517,040	0.164	0.328
106	4,453,749	464,531	0.2284	0.4568
107	4,457,649	434,769	0.328	0.656
108	4,453,960	433,319	0.2688	0.5376
